# Ebola wreaks havoc in Sierra Leone

**DOI:** 10.1186/2049-9957-4-10

**Published:** 2015-01-26

**Authors:** Mohamed Koroma, Shan Lv

**Affiliations:** Fourah Bay College, University of Sierra Leone, Freetown, Sierra Leone; Freetown City Council, Freetown, Sierra Leone; National Institute of Parasitic Diseases, China CDC, Shanghai, People’s Republic of China; WHO Collaborating Center for Malaria, Schistosomiasis and Filariasis, Shanghai, People’s Republic of China

**Keywords:** Ebola virus disease, Outbreak, Control, Measures, Government

## Abstract

**Background:**

Ebola virus disease has taken a toll on more than 8,000 lives in West Africa in 2014. The most affected countries are Guinea, Liberia, and Sierra Leone. The number of people infected by Ebola in Sierra Leone surpassed that of Liberia in the last month in this year and almost half of human cases are distributed in this country.

**Discussion:**

The ignorance on Ebola among people, including health workers at the early stage, plaid an important role in spread of Ebola virus disease. Subsequently, Ebola ravages urban settings for the first time and takes a huge toll on the lives. The government and international partners do make efforts to control the epidemic, however, lack of synergy make them lip service.

**Summary:**

The leading role of government in the response to the epidemic should be emphasized. Basic information of Ebola should be quickly spread among communities by health education programme and social mobilization should be a basic measure for Ebola control.

**Electronic supplementary material:**

The online version of this article (doi:10.1186/2049-9957-4-10) contains supplementary material, which is available to authorized users.

## Multilingual abstracts

Please see Additional file 1 for translations of the abstract into the six official working languages of the United Nations.

## Background

Ebola virus disease (EVD) has caused more than 8,000 deaths in West Africa in the past year. The most affected countries are Guinea, Liberia, and Sierra Leone [1]. This is the largest Ebola outbreak since 1976 when the virus was first discovered [2, 3]. Although the first case of the present outbreak was discovered in Guinea, the incidence in this country is at a relatively lower level as compared to Liberia and Sierra Leone. At the beginning of September 2014, the Ebola epidemic has turned the corner in Liberia, but unfortunately, there is no sign to indicate its decline in Sierra Leone. Here, we present our opinion of the current situation in Sierra Leone based on our observations from the field.

## Discussion

### Denial or ignorance: What is what as Ebola takes its toll?

The outbreak of the EVD in the Kailahun district in Eastern Sierra Leone in May 2014 is a result of a socioeconomic, religious, cultural, and political accident. A well-known and widely-respected Sierra Leonean herbalist went to the Republic of Guinea to dispense herbs to a sick person who turned out to be an Ebola victim and eventually died. The herbalist returned to Sierra Leone and fell sick; she also died and was given an honorable burial as traditional protocols demand. Hundreds of mourners came from nearby towns, which resulted in as many as 365 deaths being linked to the funeral and, in turn, triggered the subsequent Ebola epidemic in the country [4].

The people of the Kailahun district and some sections of the population thought that a plague of some sort was wrecking havoc on people’s lives. They attributed this to an attempt by the Sierra Leone government to eliminate a section of the opposition population in the country. When the virus started infecting people in the North, they attributed it to witchcraft. When it eventually emerged in the capital city of Freetown and the West, it was attributed to well water poisoning.

**Figure 1:**
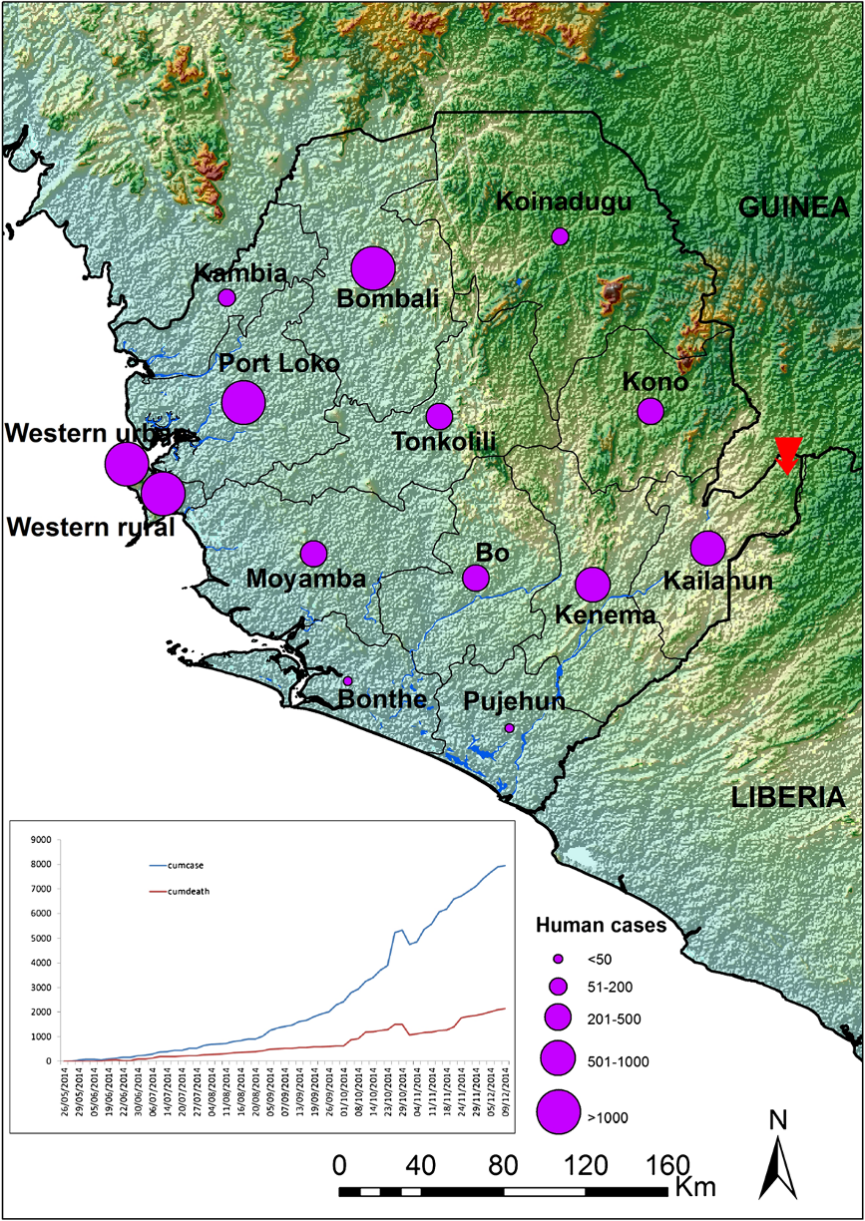
**The present situation of the Ebola virus disease in Sierra Leone.** The data are available as of December 9, 2014, as per the situation report from the Ministry of Health and Sanitation and the World Health Organization (WHO). The red arrow indicates the first case imported cross-border between Guinea and Sierra Leone.

As of December 24, the number of people infected with Ebola in Sierra Leone far surpassed that of Guinea and Liberia, resulting in the loss of 8% of the total number of doctors, with 107 deaths out of the 138 infected doctors and nurses. It is estimated that more than 20,000 people have been infected in West Africa, with almost half of these in Sierra Leone [5]. The breakdowns of the epicenter districts are as follows: Moyamba (309), Port Loko (1,574), Western Area (3,782), and Bombali (1,123) [6] (see Figure 1). Inversely, the initially hard-hit districts of Kenema and Kailahun now record 0 or one-digit cases.

### What has Sierra Leone and the international community done right and wrong in order to curb the Ebola virus disease?

Firstly, it must be noted that all partners involved in curbing the EVD are now poised with a common interest to eliminate the disease which has become an international health emergency. But even though the National Ebola Response Center (NERC) has been set up to coordinate the activities of all those fighting Ebola and there is a meeting almost every day, there seems to be lip service than actual work being done.

Our assertion is backed up by the assumption that the partners are not playing as a team, the government continues to shift the goal post, and the rules are being changed in the middle of the game, resulting in Sierra Leone being the highest Ebola infected country in the Mano River Union.

Well-placed people in governance say that changes are affected in line with the dynamics of the virus. Names and positions of the same personnel shouldn’t keep changing. We have very weak pillars in the fight, for example, contact tracing, burial teams, social mobilization, and laboratory service, etc. These pillars must be revamped and armed with the right personnel and logistic plans. Together, they should become the hub around which every partner must revolve if there is to be a meaningful breakthrough in eliminating the virus. At present, the partners seem to be flying their own kites with different altitudes resulting in no synergy in the fight.

In the midst of all of these, the approach of the Sierra Leone people in the control of Ebola can be described as a bag of nuggets and coal. There seems to be lull in the infection rate in the initially affected districts of Kenema and Kailahun mainly because the people in these districts now abide by the guidelines given by the Ministry of Health and Sanitation. But what seems to be nurturing the virus in the newly affected districts is blind passion for loved ones, ignorance about the virus’ ability to infect several people from just one infected source, and other religious, cultural, and metaphysical factors.

**Figure 2:**
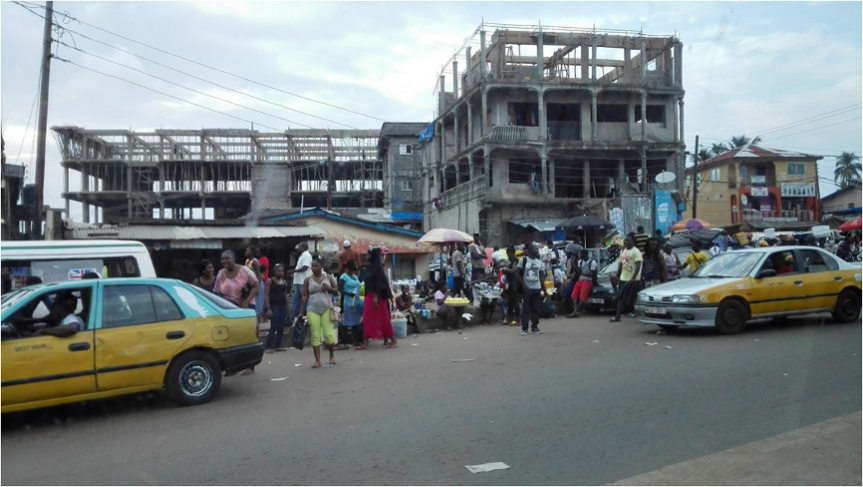
**Unaffected life in Freetown, the most affected area in Sierra Leone.**

In the west of Sierra Leone, regarded as the most informed region, there are still mass gatherings of people in market places (see Figure 2), birthday parties are still being celebrated, and funeral rites are still being performed without the slightest panic over the contagious nature of the disease. These factors are all contributing to the virus spreading.

In the north of Sierra Leone, metaphysical reasons such as witchcraft are given to explain the disease. Some believe that there was a plane crash full of witches and that is why people are dying, or exhuming and dismembering the Ebola infested bodies.

### How can Sierra Leone eliminate Ebola?

In spite of the poor funding and weak manpower, the medical community in Sierra Leone seems to be doing a marvelous job in improving the survival of the Ebola infected patients. The crude mortality (28.7% as of December 24) of the human Ebola virus infection is persistently lower in Sierra Leone as compared to other African countries that bear the burden of the disease. Therefore, the epidemic can be contained in the near future if local and international resources are used concertedly.

We agree with Sierra Leone’s president that we have a “public health emergency” on our hands. The way and manner in which Ebola is killing people is an extraordinary experience that needs extraordinary measures [7]. The perceived measures have been proclaimed by the president and approved by parliament, but not holistically enforced, as social mobilization is not being carried out sufficiently. One thing the government could do is let every Sierra Leonean know that he/she will pay a costly price if traditional funeral rites are performed on any dead body for at least the next three months.

The Government of Sierra Leone is encouraged to establish a basket fund that collects all the funds directed to curb Ebola in the country. Withdrawals of these monies should be co-signed by the government and the contributing countries according to how much they contributed. This measure will not only allow the contributing countries to decide how their funds are managed, but also support the Sierra Leone government in taking the lead in maintaining what is needed to stop the transmission of Ebola in the country.

## Summary

The West African outbreak of EVD is prevailing in urban settings and thus results to unprecedented toll on lives. Almost half of human cases are distributed in Sierra Leone. The people’s ignorance on the disease as well as the lack of synergy in governments and international organizations are contributing to the current epidemic. Therefore, the key messages of EVD should be quickly spread to communities by health education programmes and the national government should play the essential role in control of EVD and put forward robust measures to fighting against EVD.

## Electronic supplementary material

Additional file 1: **Multilingual abstracts in the six official working languages of the United Nations.** (PDF 228 KB)
