# Supplementary material for: Ebola wreaks havoc in Sierra Leone
Source: Infect Dis Poverty. 2015 Jan 26;4(1):10. doi: 10.1186/2049-9957-4-10 (PMC4328734; doi:10.1186/2049-9957-4-10)
Supplement: Supplementary file 1 — Additional file 1: Multilingual abstracts in the six official working languages of the United Nations. (PDF 228 KB) [file 40249_2014_90_MOESM1_ESM.pdf]

Translation of the abstract into the six official working languages of the United Nations

إيبولا يبث الفوضى في سيراليون

محمد كرما وشان ل في

ملخص

معلومات أساسية: لقد حصد مرض إيبولا أكثر من 8000 من الأرواح البشرية في غرب إفريقيا سنة 2014. وتعتبر الدول الأكثر تضررا هي غينيا وليبيريا وسيراليون. وقد كان عدد المصابين بفيروس إيبولا في سيراليون أكثر من عددهم في ليبيريا خلال الشهر الأخير من هذه السنة. وقد كانت نصف الحالات البشرية منتشرة في هذا البلد.

نقاش: إن الجهل بمرض إيبولا في مراحله المبكرة من قبل الناس ،بما فيهم العاملين في قطاع الصحة، لعب دورا كبيرا في انتشار المرض. لاحقا، وللمرة الأولى يستشري مرض إيبولا في المناطق الحضرية ويحصد خسائر فادحة في الأرواح. وقد قامت الحكومة وشركائها الدوليين ببذل جهود من أجل السيطرة على الوباء. لكن غياب تضافر الجهود جعل منها جهودا رمزية.

خلاصة: يجب أن يتأكد الدور الرائد للحكومة كردة فعل تجاه هذا الوباء. كما يجب أن يكون النشر السريع للمعلومات الأساسية حول مرض إيبولا بين المجتمعات ،من خلال برنامج التربية الصحية والتعبئة الإجتماعية ،هي الإجراءات الأساسية للحد من انتشار إيبولا.

Translated from English version into Arabic by malika2012, through

TRANSLATORS
WITHOUT BORDERS

埃博拉病毒病在塞拉利昂肆虐

Mohamed Koroma, Shan Lv

摘要

引言：埃博拉病毒病 2014 年爆发在西非造成 8000 多人死亡。受疫情影响最严重的国家包括几内亚、利比里亚、塞拉利昂。在 2014 年的最后一个月塞拉利昂的患者数量超过了利比里亚，目前几乎一半的病例分布在塞拉利昂。

讨论：人们（包括早期的医务工作者）对埃博拉的无知是导致该病扩散的重要原因。随后，埃博拉在城市地区的传播导致大量死亡。政府和国际组织确实在控制疫情中做了很多努力，但成效并不显著。

总结：在应对疫情中政府应该起主导作用。埃博拉基础知识应通过健康教育项目在社区中迅速推广，社会动员应当作为埃博拉控制的基础措施。

Translated from English version into Chinese by Lv Shan

Les ravages d’Ebola en Sierra Leone

Mohamed Koroma, Shan Lv

Résumé

Contexte: La maladie due au virus Ebola a fait plus de 8.000 victimes en Afrique de l'Ouest en 2014. Les pays les plus touchés sont la Guinée, le Liberia et la Sierra Leone. Le nombre de personnes infectées par Ebola en Sierra Leone a dépassé celui du Liberia au cours du dernier mois de cette année, et près de la moitié des cas humains sont répartis dans ce pays.

Discussion: l'ignorance des gens sur Ebola, y compris du personnel de santé à un stade précoce, a joué un rôle important sur la propagation de la maladie due au virus Ebola. Ensuite, Ebola a fait des ravages dans les milieux urbains pour la première fois, et causé un nombre impressionnant de victimes. Le gouvernement et les partenaires internationaux ont vraiment fait des efforts pour contrôler l'épidémie, cependant le manque de synergie les a empêchés de concrétiser ces efforts.

En résumé: le rôle directeur du gouvernement en réponse à l'épidémie devrait être mis en lumière. L'information de base sur Ebola devrait être rapidement répandue à travers les communautés par un programme d'éducation sur la santé, et une mobilisation sociale devrait être la mesure de base pour le contrôle d'Ebola.

Translated from English version into French by Ode Laforge, through

TRANSLATORS
WITHOUT BORDERS

Эбола сеет хаос в Сьерра-Лионе

Мохамед Корома, Шан Лв

Краткое описание

Базовая информация: Вирус Эбола уже унес свыше 8000 жизней в Западной Африке в 2014 году. Больше всего от заболевания пострадали такие страны, как Гвинея, Либерия и Сьерра-Лионе. В прошлом месяце число инфицированных вирусом «Эбола» в Сьерра-Лионе превысило количество заболевших в Либерии, при этом почти половина случаев инфицирования человека также приходится на эту страну.

Обсуждение: Важное значение при распространении вируса Эбола играет неосведомленность населения об этом заболевании, включая работников здравоохранения, которые не могут распознать заболевание на ранних стадиях. В результате Эбола, прежде всего, поражает городские поселения и уносит множество жизней. Правительство и международные партнеры приняли определенные меры для контроля эпидемии, однако, отсутствие совместной деятельности оказывает им «медвежью услугу».

Краткий обзор: Следует обращать большее внимание на ведущую роль правительства в рамках противодействия эпидемии. Необходимо обеспечивать быстрое распространение информации об Эболе среди сообществ с помощью образовательных программ в сфере здоровья и социальной мобилизации, которые должны являться основной мерой контроля распространения этого вируса.

Translated from English version into Russian by Irina Zayonchkovskaya, through

TRANSLATORS
WITHOUT BORDERS

El ébola causa estragos en Sierra Leona

Mohamed Koroma, Shan Lv

Introducción

Información de referencia: La enfermedad del virus del ébola se ha cobrado más de 8.000 vidas en África Occidental durante 2014. Los países más afectados son Guinea, Liberia y Sierra Leona. El número de personas infectadas por el ébola en Sierra Leona superó al de Liberia durante el mes pasado de este año y casi la mitad de los casos en humanos se distribuyen por este país.

Debate: La ignorancia sobre el ébola entre las personas, incluyendo al personal sanitario en fase inicial, jugó un importante papel en la propagación de la enfermedad del virus del ébola. Como consecuencia de ello, el ébola está devastando a las poblaciones urbanas por primera vez y se está cobrando un gran número de vidas. El gobierno y los colaboradores internacionales dedicaron esfuerzos a controlar la epidemia; sin embargo, la falta de sinergias hizo que todo se quedara en buenas palabras.

Resumen: Es necesario enfatizar el liderazgo que ha de desempeñar el gobierno en respuesta a la epidemia. Se debería distribuir rápidamente entre las comunidades la información sobre el ébola a través de programas de educación sanitaria y movilización social como medida básica para controlar el ébola.

Translated from English version into Spanish by Sergio Lorenzi, through

TRANSLATORS
WITHOUT BORDERS
